# Reviewer summary for Journal of Arrhythmia

**DOI:** 10.1002/joa3.13063

**Published:** 2024-06-10

Chang, Shih‐Lin

slchang4@vghtpe.gov.tw


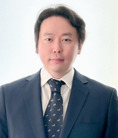


Hojo, Rintaro

rinrintaro1979@hotmail.co.jp


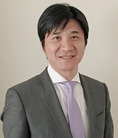


Morishima, Itsuro

morishima-i@muc.biglobe.ne.jp


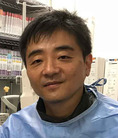


Nakahara, Shiro

nshiro@dokkyomed.ac.jp


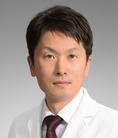


Naruse, Yoshihisa

ynaruse@hama-med.ac.jp


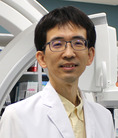


Yoshida, Kentaro

kentaroyo@nifty.com

Arimoto, Takanori

Fukaya, Hidehira

Morita, Norishige

Shimizu, Akihiko

Aizawa, Yoshiyasu

Nishii, Nobuhiro

Heeger, Christian

Inden, Yasuya

Kumagai, Koji

Mukai, Yasushi

Ching, Chi Keong

Kobori, Atsushi

Maruyama, Mitsunori

Miyazaki, Shinsuke

Nakai, Toshiko

Noda, Takashi

Sobue, Yoshihiro

Yamasaki, Hiro

Chinushi, Masaomi

Horigome, Hitoshi

Ikeda, Yoshifumi

Iwasaki, Yuki

Oginosawa, Yasushi

Sato, Toshiaki

Satomi, Kazuhiro

Ueda, Akiko

Watanabe, Eiichi

Yamashita, Seigo

Yokoshiki, Hisashi

Aoki, Hisaaki

Futyma, Piotr

Ishibashi, Kohei

Kaneko, Yoshiaki

Kato, Hiroyuki

Kimura, Masaomi

Nagashima, Koichi

Sasaki, Wataru

Shizuta, Satoshi

Yoshimoto, Jun

Chung, Fa‐Po

Fujiu, Katsuhito

Fukuzawa, Koji

Harada, Masahide

Hayashi, Tatsuya

Imai, Katsuhiko

Kabutoya, Tomoyuki

Kamakura, Tsukasa

Masuda, Masaharu

Oka, Takafumi

Sekihara, Takayuki

Sumitomo, Naokata

Tokuda, Michifumi

Tsutsui, Kenta

Yagi, Tetsuo

Yoshiga, Yasuhiro

Bunch, Thomas

Hachiya, Hitoshi

Hasegawa, Kanae

Higa, Satoshi

Imai, Yasushi

Irie, Tadanobu

Kajiyama, Takatsugu

Kawamura, Mitsuharu

Komatsu, Yuki

Kuroki, Kenji

Matsumoto, Kazuhisa

Matsuo, Seiichiro

Miyauchi, Yasushi

Miyazaki, Aya

Nagashima, Michio

Nakajima, Kenzaburo

Nodera, Minoru

Okumura, Yasuo

Shinohara, Tetsuji

Suzuki, Shinya

Wada, Mitsuru

Yamauchi, Yasuteru

Yanagisawa, Satoshi

Amaya, Naoki

Chiladakis, John

Ejima, Koichiro

Fukuda, Koji

Fukunaga, Masato

Hayashi, Kentaro

Hsieh, Yu‐Cheng

Inoue, Yuko

Isawa, Tsuyoshi

Kodani, Eitaro

Kohno, Ritsuko

Lin, Lian‐Yu

Mitsuhashi, Takeshi

Okada, Ayako

Shirai, Yasuhiro

Suzuki, Tsugutoshi

Takahashi, Yoshihide

Takatsuki, Seiji

Tobiume, Takeshi

Tsuboi, Ippei

Watanabe, Atsuyuki

Yamaguchi, Takanori

Akao, Masaharu

Akima, Takashi

Arita, Takuto

Asano, Taku

Calkins, Hugh

Chen, Wei‐Ta

Fujino, Tadashi

Fukamizu, Seiji

Furusho, Hiroshi

Hosoda, Junya

Inoue, Koichi

Joung, Boyoung

Kasai, Takatoshi

Kato, Takeshi

Kawakami, Hiroshi

Kawamura, Iwanari

Kutarski, Andrzej

Lee, Jong‐Kook

Liao, Jo‐Nan

Liao, Ying‐Chieh

Maruyama, Toru

Miyanaga, Satoru

Mizutani, Yoshiaki

Mori, Hitoshi

Murata, Hiroshige

Nagase, Satoshi

Nakamura, Toshihiro

Narita, Masataka

Ngarmukos, Tachapong

Niwano, Shinichi

Ogano, Michio

Ohe, Masatsugu

Ohno, Seiko

Ono, Katsushige

Osanai, Hiroyuki

Sasaki, Shingo

Sasaki, Takeshi

Shiga, Tsuyoshi

Shinohara, Masaya

Soeki, Takeshi

Stabile, Giuseppe

Takeuchi, Daiji

Tao, Susumu

Terata, Ken

Tokutake, Ken‐Ichi

Toyohara, Keiko

Tsuji, Yukiomi

Yamabe, Hiroshige

Yodogawa, Kenji

Aizawa, Yoshifusa

Ashihara, Takashi

Enjoji, Yoshihisa

Enomoto, Yosinari

Gatzoulis, Konstantinos

Hayashi, Kenshi

Hayashi, Meiso

Higuchi, Satoshi

Hiroshima, Ken‐Ichi

Hong, Kui

Hori, Yuichi

Hu, Yu‐Feng

Ishizue, Naruya

Kataoka, Naoya

Kato, Yoshiaki

Kokubo, Yoshihiro

Kumagai, Koichiro

Kuroda, Shunsuke

Lee, Kun‐Tai

Lee, Pi‐Chang

Lin, Wei‐Shiang

Mar, Philip L.

Matsumoto, Naoki

Minamiguchi, Hitoshi

Mine, Takanao

Miyamoto, Koji

Miyazaki, Yuichiro

Mizukami, Akira

Murakami, Masato

Nakamura, Kazufumi

Nakamura, Tomofumi

Nozoe, Masatsugu

Oka, Satoshi

Okubo, Yousaku

Otsuki, Sou

Sairaku, Akinori

Sheunnnan, Chiu

Suzuki, Makoto

Takagi, Masahiko

Tanabe, Yasuko

Tonegawa‐Kuji, Reina

Tsurugi, Takuo

Wakamiya, Akinori

Watanabe, Masaya

Wilde, Arthur

Yamada, Shinya

Yashima, Masaaki

Yazaki, Kyoichiro

Yuniadi, Yoga

Zou, Fengwei

An, Yoshimori

Bun, Sok‐Sithikun

Canpolat, Uğur

Cha, Myung‐Jin

Chao, Tze‐Fan

Coner, Ali

Frontera, Antonio

Fukui, Akio

Gupta, Dhiraj

Hachisuka, Masato

Hasebe, Hideyuki

Hashimoto, Kenichi

Hayashi, Hiroshi

Hung, Chung‐Lieh

Ikeda, Takanori

Inaba, Osamu

Inamura, Yukihiro

Ito‐Hagiwara, Kanako

Jędrzejczyk‐Patej, Ewa

Karadeniz, Cem

Kawabata, Mihoko

Kawano, Hiroyuki

Kondo, Hidekazu

Kondo, Yusuke

Kuan‐Hung, Yeh

Kurokawa, Sayaka

Kuwahara, Taishi

Lin, Gen‐Min

Lin, Wen‐Yu

Lo, Li‐Wei

Makiyama, Takeru

Mari, Amino

Marine, Joseph E. E.

Matsunaga‐Lee, Yasuharu

Menichelli, Danilo

Miyagi, Yasuo

Nabeshima, Taisuke

Nakamura, Keijiro

Nishiyama, Nobuhiro

Oh, Il‐Young

Oh, Yong‐Seog

Romiti, Giulio Francesco

Sasano, Tetsuo

Sekiguchi, Yukio

Soejima, Kyoko

Sugai, Yoshinao

Sugiyama, Atsushi

Takahashi, Masao

Takemoto, Masao

Takigawa, Masateru

Tanaka, Nobuaki

Tanno, Kaoru

Teo, Wee‐Siong

Tisdale, James E.

Togashi, Ikuko

Tokano, Takashi

Tsai, Chia‐Ti

Tsai, Chin‐Feng

Usuda, Keisuke

Yada, Hirotaka

Yagishita, Atsuhiko

Yamagata, Kenichiro

Yamaji, Hirosuke

Yasuoka, Ryobun

Yokoyama, Yasuhiro

Yoshida, Yoko

Yoshioka, Koichiro

Yu, Chih‐Chieh

Yuzawa, Hitomi

